# AsnB Mediates Amidation of *Meso*-Diaminopimelic Acid Residues in the Peptidoglycan of *Listeria monocytogenes* and Affects Bacterial Surface Properties and Host Cell Invasion

**DOI:** 10.3389/fmicb.2021.760253

**Published:** 2021-10-15

**Authors:** Lei Sun, Gil Rogiers, Pascal Courtin, Marie-Pierre Chapot-Chartier, Hélène Bierne, Chris W. Michiels

**Affiliations:** ^1^Laboratory of Food Microbiology, Department of Microbial and Molecular Systems (M2S) and Leuven Food Science and Nutrition Research Center (LFoRCe), KU Leuven, Leuven, Belgium; ^2^INRAE, AgroParisTech, Micalis Institute, Université Paris-Saclay, Jouy-en-Josas, France

**Keywords:** *Listeria monocytogenes*, *meso*-diaminopimelic acid, peptidoglycan modification, virulence, motility, biofilm formation, lysozyme sensitivity, host cell invasion

## Abstract

A mutant of *Listeria monocytogenes* ScottA with a transposon in the 5' untranslated region of the *asnB* gene was identified to be hypersensitive to the antimicrobial *t*-cinnamaldehyde. Here, we report the functional characterization of AsnB in peptidoglycan (PG) modification and intracellular infection. While AsnB of *Listeria* is annotated as a glutamine-dependent asparagine synthase, sequence alignment showed that this protein is closely related to a subset of homologs that catalyze the amidation of *meso*-diaminopimelic acid (*m*DAP) residues in the peptidoglycan of other bacterial species. Structural analysis of peptidoglycan from an *asnB* mutant, compared to that of isogenic wild-type (WT) and complemented mutant strains, confirmed that AsnB mediates *m*DAP amidation in *L. monocytogenes*. Deficiency in *m*DAP amidation caused several peptidoglycan- and cell surface-related phenotypes in the *asnB* mutant, including formation of shorter but thicker cells, susceptibility to lysozyme, loss of flagellation and motility, and a strong reduction in biofilm formation. In addition, the mutant showed reduced invasion of human epithelial JEG-3 and Caco-2 cells. Analysis by immunofluorescence microscopy revealed that *asnB* inactivation abrogated the proper display at the listerial surface of the invasion protein InlA, which normally gets cross-linked to *m*DAP *via* its LPXTG motif. Together, this work shows that AsnB of *L. monocytogenes*, like several of its homologs in related Gram-positive bacteria, mediates the amidation of *m*DAP residues in the peptidoglycan and, in this way, affects several cell wall and cell surface-related properties. It also for the first time implicates the amidation of peptidoglycan *m*DAP residues in cell wall anchoring of InlA and in bacterial virulence.

## Introduction

Bacterial cells are surrounded by a rigid peptidoglycan (PG) cell wall whose primary universal function is to maintain cell shape and preserve cell integrity, particularly in hypo-osmotic environments that would otherwise be conducive to cell lysis (Vollmer et al., 2008). The tensile strength required for this function is derived from the mesh-like structure of PG, which consists of long polymeric chains of N-acetylglucosamine (GlcNAc), N-acetylmuramic acid (MurNAc) heterodisaccharides, and cross-linked *via* peptide side chains. While this basic architecture has been well conserved, there is a wide variety in PG chemical structures in different bacteria. This variation stems mainly from differences in the amino acid composition and cross-linking of side chains but also from the presence – or removal – of modifying substituents on the sugar and amino acid units (Yadav et al., 2018). Differences in PG structure have also been observed depending on the growth stage and growth conditions in several bacteria and are developmentally regulated in spore-forming bacteria, where a distinct and unique δ-lactam PG modification exists in the so-called spore cortex, a thick protective PG layer surrounding the germ cell wall which lacks this modification. This allows specific hydrolases embedded on the spore’s surface to selectively cleave the cortex without compromising the germ cell wall upon spore germination.

Besides having a structural role, PG is also a scaffold anchoring surface proteins with various functions (Bierne and Cossart, 2007), and PG or PG fragments also serve as a molecular signal in several symbiotic or pathogenic interactions with animal or plant hosts (Gust, 2015; Irazoki et al., 2019). The high specificity required for such signaling is made possible by the presence of some unique or rare building blocks including MurNAc, *meso*-diaminopimelic acid (*m*DAP) and D-amino acids, and by the large structural variation in PG which is generated in part by enzymatic modifications. PG modifications are widespread in Gram-positive and Gram-negative bacteria, and are often important for virulence in pathogens (Yadav et al., 2018). N-deacetylation of GlcNAc, for example, occurs in pathogens including various streptococci and *Listeria monocytogenes*, prevents PG hydrolysis by lysozyme, and helps cells to evade the immune response during infection (Vollmer and Tomasz, 2000; Boneca et al., 2007; Fittipaldi et al., 2008). The most widespread and best studied modification is probably O-acetylation of MurNAc, and this modification has also been implicated in lysozyme resistance and virulence in pathogens like *Neisseria gonorrhoeae* and *Staphylococcus aureus* (Brott and Clarke, 2019). Even more variations exist in the stem peptide as compared to the sugar backbone. These include the addition of substituents, with the most widespread and best documented examples being the amidation of D-*iso*-Glu and *m*DAP residues, on their α-carboxyl and ε-carboxyl groups, respectively. These amidation modifications occur in several genera and species of Gram-positive bacteria, while the only example reported in Gram-negative bacteria thus far is the amidation of the α-carboxyl group of *m*DAP in *Acetobacteraceae* (Espaillat et al., 2016).

Stem peptide amidation has been reported to affect different bacterial properties and functions. The amidation of D-*iso*-Glu is mediated by MurT/GatD and is essential in *S. aureus* and *Streptococcus pneumoniae*, and reduced expression of the *murT*/*gatD* operon enhanced the sensitivity to β-lactam antibiotics and lysozyme in *S. aureus* (Figueiredo et al., 2012; Liu et al., 2017). The ε-carboxyl amidation of *m*DAP is mediated by an enzyme that is homologous to the glutamine-dependent asparagine (Asn) synthetase found in various organisms, such as AsnB of *Escherichia coli*. At least some of these enzymes are promiscuous, being able to catalyze the amidotransfer from glutamine not only to aspartate for the synthesis of Asn, but also to *m*DAP for modification of the PG stem peptide. *Bacillus subtilis* encodes three homologs, designated AsnB, AsnO, and AsnH, and while each of these can complement the Asn deficiency of an *E. coli* Asn auxotroph, none of them is essential for Asn synthesis in *B. subtilis*, since even a triple knockout mutant could still grow without Asn, albeit at a reduced rate (Yoshida et al., 1999). It was later shown that AsnB, but neither of its two homologs, mediates *m*DAP amidation (Dajkovic et al., 2017). Furthermore, *asnB* was essential unless excess Mg^2+^ was provided in the growth medium, and its deletion rendered cells sensitive to antibiotics targeting the cell wall and to lysozyme (Dajkovic et al., 2017). *Lactiplantibacillus plantarum* (former name *Lactobacillus plantarum*) has two homologs, AsnB1 and AsnB2, of which the former was shown to mediate *m*DAP amidation (Bernard et al., 2011). Mutants in which AsnB1 was deactivated were affected in growth and showed filamentation, suggesting a role of amidation in the cell septation process. In addition, amidation also controlled the activity of the L,D-carboxypeptidase DacB that trims the stem peptide. A similar situation – two homologs of which one specifically mediates *m*DAP amidation – exists in *Clostridioides difficile*, but a remarkable feature in this organism is that the expression of the *asnB* gene that confers *m*DAP amidation is specifically induced by vancomycin (Ammam et al., 2020). Somewhat unexpectedly, amidation slightly reduced vancomycin resistance but did not affect lysozyme resistance, and its role in *C. difficile* therefore remains unclear. While *B. subtilis*, *L. plantarum*, and *C. difficile* belong to the *Firmicutes* (low GC) phylum of Gram-positive bacteria, an AsnB homolog mediating *m*DAP amidation has also been identified in *Corynebacterium glutamicum* in the (high GC) *Actinobacteria* phylum (Levefaudes et al., 2015). *Corynebacterium glutamicum* encodes only one AsnB homolog (designated LtsA), and deletion of the gene resulted in loss of *m*DAP amidation, attenuated growth, morphological changes, and sensitivity to cell wall-targeting antibiotics and lysozyme, thus mirroring the phenotype of an *asnB* knockout mutant in *B. subtilis*. Mycobacteria, finally, also belong to the *Actinobacteria* and have amidated *m*DAP residues as well. Although there is no direct evidence for a role of their AsnB homologs in *m*DAP amidation, knockout mutants have been isolated and partially characterized. In *Mycobacterium smegmatis*, a transposon insertion mutant in *asnB* showed a delayed onset of growth and displayed sensitivity to multiple antibiotics (Ren and Liu, 2006). In *Mycobacterium tuberculosis*, an *in vitro* enzymatic assay demonstrated that *m*DAP amidation was required for the formation of cross-links between neighboring stem peptides by the L,D-transpeptidase Ldt, and construction of a conditional *asnB* knockout led to the conclusion that the gene was essential (Ngadjeua et al., 2018).

In the present work, we report the isolation of an *asnB* mutant from a screen of a genome-wide *L. monocytogenes* transposon insertion library against the natural antimicrobial *t*-cinnamaldehyde (*t*-CIN). *Listeria monocytogenes* is a foodborne pathogen that occasionally causes systemic infections with a high mortality rate, primarily in immunocompromised individuals and the fetus of pregnant women. The organism is widespread and thrives in a variety of terrestrial and aqueous natural environments, as well as in man-made environments like food production plants. Moreover, it can also engage in a highly specialized and complex pathogenic interaction with the human host or diverse animal species. In particular, *L. monocytogenes* can invade non-phagocytic mammalian cells through different steps, including entry into the host cell by receptor-mediated endocytosis, escape from the entry vacuole, bacterial replication in the cytoplasm, actin-based motility that allows for cell-to-cell spread (Radoshevich and Cossart, 2018), and, in some cell types, persistence in vacuoles (Bierne et al., 2018). Like many other *Bacilli*, *L. monocytogenes* PG contains *m*DAP in the third position of the stem peptide, and at least in some strains, the residue is also amidated (Boneca et al., 2007; Burke et al., 2014). However, while various PG modifications have been implicated in virulence in several pathogens, including N-deacetylation and O-acetylation in *L. monocytogenes* (Boneca et al., 2007; Aubry et al., 2011), a similar role has not yet been demonstrated for *m*DAP amidation in any pathogen. Therefore, the goal of this study was to conduct a detailed functional analysis of AsnB in *L. monocytogenes*. Our results confirm that AsnB catalyzes *m*DAP amidation and provide evidence for a role in cell wall homeostasis, flagellum-mediated motility, and bacterial pathogenicity.

## Materials and Methods

### Bacterial Strains and Plasmid Construction

The bacterial strains and plasmids used in this work are listed in Table 1. *Listeria monocytogenes* Scott A was used as the wild-type (WT) strain and acquired from the International Life Sciences Institute (ILSI) North America (Fugett et al., 2006). Strain *asnB::Himar1* was identified as a sensitive mutant from a random Scott A transposon mutant collection in a screen with the natural antimicrobial *t*-CIN (Rogiers et al., 2017). *Escherichia coli* DH5α (Grant et al., 1990) was employed as host for cloning constructs, and *E. coli* S17-1 λpir (Simon et al., 1983) as the donor strain for conjugational plasmid transfer. *Listeria monocytogenes* strains were grown at 30 or 37°C in Brain Heart Infusion (BHI, Oxoid, Hampshire, United Kingdom) medium. *Escherichia coli* strains were grown in Luria-Bertani (LB, 10-g/L tryptone, 5-g/L yeast extract, and 5-g/L NaCl) medium at 37°C. Growth media were supplemented with 50-μg/ml erythromycin (Em; Acros Organics, Fair Lawn, NJ, United States) or 50-μg/ml kanamycin (Km; AppliChem GmbH, Darmstadt, Germany) when appropriate.

**Table 1:** Strains and plasmids in this work.

Bacterial species	Designation in this work	Description/Construction	References
*Lmonocytogenes*	*WT*	wild type strain Scott A	Fugett et al., 2006
	*WT/pIMK2*	WT with pIMK2 integrated, Km^R^	This work
	*asnB::Himar1*	Transposon insertion at 5' end of *asnB*, Em^R^	This work
	*asnB/pIMK2*	*5’ asnB::Himar1* with pIMK2 integrated, Km^R^ Em^R^	This work
	*asnB/pIMK2-asnB*	*5’ asnB::Himar1* with pIMK2-asnB integrated, Km^R^ Em^R^	This work
*E. coli*	S17-1 λpir	Donor for plasmid conjugation	Simon et al., 1983
	DH5-α	Host strain for plasmid constructs	Grant et al., 1990
**Plasmids**	**Description**		**References**
pIMK2	Site-specific listerial integrative vector, pHelp promoter for constitutive overexpression, 6.2kb, Km^R^		Monk et al., 2008
pIMK2-*asnB*	pIMK2 with *asnB* gene from Scott A under control of pHelp promotor		This work

For genetic complementation of the *asnB::Himar1* mutant, the *asnB* gene was amplified using primers asnB_NcoI and asnB_SalI (Table 2) and cloned in pIMK2 (Monk et al., 2008) digested with NcoI and SalI. The construct was verified with Sanger sequencing and then conjugated from *E. coli* S17-1 λpir into the *asnB::Himar1* mutant. Successful integration was confirmed *via* PCR with primers asnB_NcoI and NC16 (which anneals near, and points toward, the plasmid integration site) and Sanger sequencing with primers pIMK_FW and pIMK_REV, which point toward the *asnB* gene from both sides of the pIMK2 cloning site. The complemented strain was designated *asnB/pIMK2-asnB*. Control strains were constructed by integration of the empty pIMK2 plasmid into the WT and *asnB::Himar1* strains and were designated as *WT/pIMK2* and *asnB/pIMK2*, respectively.

**Table 2:** Primers used in this work.

Primer	Sequence (5'–3')^*^	References
asnB_NcoI	GCATCCATGGGATGTGGATTTGTAGGATGCGTAC	This work
asnB_SalI	CACTGTCGACTTATTTTCCAAAATCGTATTTATCTGC	This work
pIMK_REV	CCTATCACCTCAAATGGTTCG	Rogiers et al., 2017
pIMK_FW	GAGTCAGTGAGCGAGGAAGC	Rogiers et al., 2017
NC16	GTCAAAACATACGCTCTTATC	Rogiers et al., 2017
Ylinker	CTGCTCGAATTCAAGCTTCT	Rogiers et al., 2017
Marq269	GCTCTGATAAATATGAACATGATGAGTGAT	Rogiers et al., 2017

* *Restriction sites: NcoI (CCATGG) and SalI (GTCGAC)*.

### Growth Assay

Growth curves of strains *WT/pIMK2*, *asnB/pIMK2*, and *asnB/pIMK2-asnB* were established by turbidity measurement (OD_630_) of cultures growing in an automated temperature-controlled microplate reader (Multiskan Ascent®, Thermo Fisher Scientific). Inocula were prepared by adjusting overnight 4-ml BHI broth cultures to the same turbidity (OD_600_≈2) using an Ultrospec™ 10 Cell Density Meter (Biochrom, Cambridge, United Kingdom), and then diluting the suspensions 1,000-fold in BHI or BHI with 3- or 4-mM *t*-CIN (Acros Organics). Subsequently, 200-μl aliquots were transferred into a 96-well microplate, which was then covered with a transparent adhesive foil (Greiner Bio-One, Frickenhausen, Germany) and incubated at 30 or 37°C in the microplate reader. Every 15min, the plates were shaken at 960rpm and OD630 was measured. The Excel add-in package DMFit (Quadram Institute Bioscience, Norwich, United Kingdom) was used to determine the maximum growth rate (μ_max_), the lag phase time (*λ*), and the maximal cell density (OD_max_) value at stationary phase based on the Baranyi and Roberts microbial growth model (Baranyi and Roberts, 1994).

### Sensitivity to Lysozyme

Lysozyme sensitivity was evaluated by a disk diffusion assay and broth growth inhibition assay as previously described (Rae et al., 2011) with minor modifications. For the disk diffusion assay, 5μl of an overnight culture was evenly spread on a BHI agar plate, and a 6-mm sterile Whatman paper disk was placed in the center and impregnated with 10μl of a 10-mg/ml lysozyme (Sigma Aldrich, Saint Louis, MO, United States) solution in 10-mM potassium phosphate buffer (PPB) pH 7.0. The size of the formed inhibition halo was measured after incubation at 30°C for 24h. For the broth inhibition assay, overnight cultures were diluted 1,000-fold in BHI broth supplemented with 1-mg/ml lysozyme and 200-μl aliquots of the cell suspension were loaded into 96-well microplates. The OD_630_ was monitored with 15-min intervals in the microplate reader at 30°C. Additionally, a lysozyme lysis assay was conducted using cells from overnight cultures that were washed and resuspended in 10-mM PPB pH 7.0 to an OD_450_ of 0.6–0.8. Two hundred and seventy microliter of the suspension were then dispensed into a 96-well microplate and 30μl of a 1,000-fold diluted lysozyme stock (1mg/ml or water for control) was added. The OD_450_ was recorded with 30-min intervals in the microplate reader at 25°C.

### Flagellar Staining and Swimming Motility Assay

Overnight stationary cultures were diluted 100-fold in BHI medium and grown to exponential phase (2–3h) at 30°C with shaking. Crystal violet flagellar staining was performed as described (Upadhyay et al., 2012), and cells were observed with a Leica SFL4000 microscope. Swimming motility was evaluated by picking colonies from a BHI plate and stab-inoculating them into BHI soft agar (0.2%) with a toothpick (Gründling et al., 2004). Plates were incubated at 30°C for 24h, and motility was assessed by measuring the migration distance of bacteria from the center to the periphery of the colony.

### High-Resolution Scanning Electron Microscopy and Cell Dimension Measurement

Cells were harvested from BHI broth at OD_600_≈1, washed once with 0.1-mM PPB pH 7.0, and diluted appropriately. Fifty microliter of cell suspension was applied to a coverslip mounted to an AI stub by carbon adhesive disks and dried in the oven at 37°C. HR SEM was performed on a Nova NanoSEM450 (FEI) scanning electron microscope.

To measure the cell dimensions, 1μl of an appropriately diluted exponential culture (OD_600_≈1) was applied to 2% agarose pads deposited on a microscopy slide. A Gene Frame (Thermo Fisher Scientific) was used to mount a cover glass on the microscopy slide. Observations were performed with an Eclipse Ti-E inverted microscope (Nikon Instruments Europe BV, Netherlands) in phase contrast modus at a total magnification of 100x and images were acquired using NIS-elements software (Nikon). Image analysis (cell width and cell length) was conducted with the MicrobeTracker software (Sliusarenko et al., 2011), with manual curation to remove false segmentation and tracking.

### Bioinformatics Analysis

The amino acid sequences of AsnB from *L. monocytogenes* Scott A and its homologs from other bacteria were acquired from the National Center for Biotechnology Information database (NCBI) and listed in Supplementary Table S1. Amino acid sequence alignment was conducted with MUSCLE (Edgar, 2004), and a phylogenic tree was constructed with CLC Genomic Workbench (QIAGEN, Hilden, Germany) using the Neighbor-joining (NJ) method with 100 bootstrap replicates.

### Peptidoglycan Extraction and Structural Analysis

PG was purified as described (Courtin et al., 2006) with minor modifications. Overnight cultures were diluted 100-fold in 0.5-L BHI broth and grown to OD_600_≈1 at 30°C. After cooling in ice water (20–30min), cells were collected by centrifugation (5,000rpm, 10min, 4°C), resuspended in 40-ml cold H_2_O, boiled (10min), cooled again, and centrifuged. After suspending the cell pellet in 1-ml H_2_O, 1-ml SDS solution (10% SDS, 100-mM Tris-HCl pH 7.0) at 60°C was added and the suspension was boiled (30min) and centrifuged (10min, 14,000rpm, RT). The pellet was resuspended in 2-ml lysis solution (4% SDS, 50-mM Tris-HCl pH 7.0), boiled (15min), and washed 6 times with H_2_O preheated to 60°C. Afterward, the pellets were treated with 2mg/ml pronase from *Streptomyces griseus* (Roche, Basel, Switzerland) in 50-mM Tris-HCl pH 7.0 for 1.5h at 60°C, and with 10-μg/ml DNase (Thermo Fisher Scientific), 50-μg/ml RNase (Thermo Fisher Scientific), and 50-μg/ml lipase from *Aspergillus niger* (Sigma Aldrich) in 20-mM Tris-HCl pH 7.0, 1-mM MgCl_2_, and 0.05% sodium azide for 4h at 37°C. Then, the suspensions were washed with H_2_O and treated with 200-μg/ml trypsin (Sigma Aldrich) in 20-mM Tris-HCl pH 8.0 at 37°C with agitation overnight. Finally, after inactivating trypsin by boiling for 3min, the suspensions were incubated with 48% hydrofluoric acid (Merck, Kenilworth, NJ, United States) overnight at 4°C. After centrifugation (10min, 14,000rpm, RT), the pellet was washed twice with 250-mM Tris-HCl (pH 7.0) and four times with H_2_O to reach a pH close to 5. The extracted PG was eventually lyophilized and resuspended in H_2_O to 20mg/ml.

For structural analysis, 50μl of purified PG was digested by adding 50-μl 25mM NaHPO_4_ pH 5.5 and 2-μl 10mg/ml mutanolysin from *Streptomyces globisporus* (Sigma Aldrich) and incubating overnight at 37°C with shaking. The resulting soluble muropeptides were reduced with sodium borohydride and separated by reverse phase-ultra high-pressure liquid chromatography (RP-UHPLC) with a 1,290 chromatography system (Agilent Technologies, Santa Clara, CA, United States) and a Zorbax Eclipse Plus C18 RRHD column (100 by 2.1mm; particle size, and 1.8μm; Agilent Technologies) at 50°C using ammonium phosphate buffer and methanol linear gradient as described previously (Courtin et al., 2006). One microliter of collected muropeptides was then spotted directly on the matrix-assisted laser desorption ionization (MALDI) target and thoroughly mixed with 1μl of α-cyano-4-hydroxycinnaminic acid solution (5mg/ml in 50% acetonitrile containing 0.1% trifluoroacetic acid). Muropeptides were analyzed by MALDI – time of flight mass spectrometry (MALDI-TOF MS) with an UltrafleXtreme instrument (Bruker Daltonics, Billerica, MA, United States; located at Université Paris-Saclay, CEA, INRAE, Médicaments et Technologies pour la Santé (MTS), MetaboHUB, Gif-sur-Yvette, France). MS spectra were acquired at 2kHz laser repetition rate in the positive reflector ion mode, with a 20-kV acceleration voltage and an extraction delay of 130ns. The spectra were obtained by accumulating 1,000–5,000 shots (depending on the samples) over the 500–5,000m/z range. MS/MS spectra were acquired in LIFT mode, at 1kHz laser repetition rate applying 7.5kV for initial acceleration of ions and 19kV for reacceleration of fragments in the LIFT cell.

### Human Cell Lines and Invasion Assays

Human JEG-3 placental cells (ATCC HTB-36) and Caco-2 intestinal cells (ATCC HTB-37) were grown following ATCC recommendations at 37°C in a humidified 10% CO_2_ atmosphere. The invasion of *L. monocytogenes* strains was assessed by the gentamicin assay, as described (Bierne et al., 2002) with some modifications. To prepare bacterial inoculums, bacterial cultures were grown to early exponential or stationary phase at 37°C, washed with 10-mM phosphate-buffered saline (PBS) pH 7.4, and diluted in Eagle’s Minimum Essential Medium (MEM). JEG-3 and Caco-2 cells at 80% confluency were infected with bacterial inoculums at a multiplicity of infection (MOI) of about 0.05–0.1 bacteria per cell and centrifuged at 300 x *g* for 2min to synchronize bacterial entry. For JEG-3 cells, after 1-h incubation, cells were washed with MEM and incubated with complete culture media containing 25-μg/ml gentamicin for 3h to kill the extracellular bacteria. For Caco-2 cells, after 0.5-h incubation, cells were washed with MEM and incubated with 25-μg/ml gentamicin for 0.5h to kill the extracellular bacteria while minimizing the intracellular replication of the infected bacteria. Subsequently, infected cells were washed twice in MEM and lysed in cold distilled water. The number of bacteria in bacterial inoculums and cell lysates was determined by serial dilutions plated on BHI agar and counting colony-forming units (cfu) after 48-h incubation at 37°C. The relative entry efficiency was expressed as the ratio of cfu recovered after cell lysis to inoculated cfu.

### Immunofluorescence Microscopy

Immunofluorescent staining was performed as described (Bierne et al., 2002). Briefly, overnight bacterial cultures were washed twice with PBS, fixed on a coverslip with 4% paraformaldehyde in PBS, and stained for 1h with a mixture of mouse monoclonal InlA antibodies L7.7 and G6.1 (Bierne et al., 2002) and a rabbit polyclonal *L. monocytogenes* antibody in 2% bovine serum albumin, at 1:500 dilution. Coverslips were then washed several times with PBS and incubated with Alexa 488-labeled goat anti-rabbit (1:400 dilution; Molecular Probes, Eugene, OR, United States) and Cy3-labeled goat anti-mouse (Jackson ImmunoResearch, West Grove, PA, United States) secondary antibodies for 1h, followed by mounting with 10μl of Fluoromount G (Interchim, Montluçon, France).

### Statistical Analysis

Data for the growth assay and intracellular infection assay are presented as means±SD from three independent repetitions. Differences of growth parameters were statistically analyzed by the Tukey’s honestly significant difference (Tukey’s HSD) test using GraphPad PRISM 7.0 (GraphPad, San Diego, CA, United States). Bacterial cell width and length are means±SD of 300 cells and the significance of mean differences was calculated by student’s *t*-test (two tailed). For the lysozyme lysis assay, linear regression was applied to fit straight lines through the data points and the slopes are presented as means±SD from five independent repetitions. The significance of mean differences was calculated by ANOVA (two-way ANOVA) using GraphPad Prism software. Values of *p*<0.05 were considered statistically significant.

## Results

### *asnB* Mutants Show Increased Sensitivity to the Antimicrobial *t*-Cinnamaldehyde and Have Altered Cell Shape

**Figure 1:**
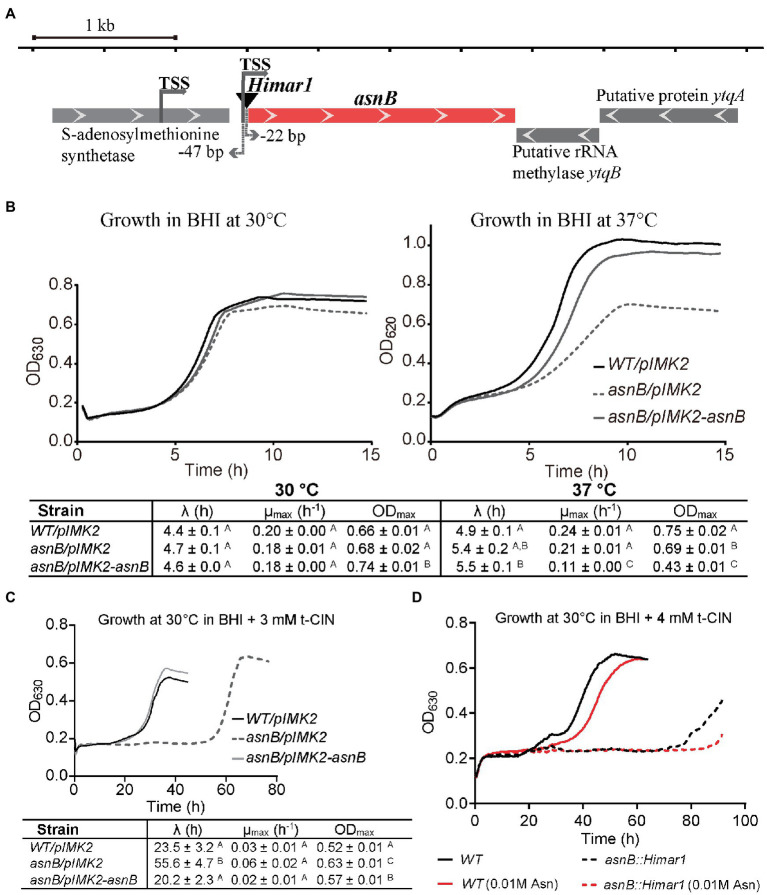
Characterization of the *t*-CIN sensitive *Listeria monocytogenes asnB* mutant. **(A)** Genomic context of the *asnB* gene. Gene orientations are indicated by white arrows in the gene boxes. The *Himar1* transposon (black inverted triangle) is inserted at −22bp of the *asnB* start codon. Transcription start sites (TSSs; Wurtzel et al., 2012) of *asnB* are indicated with gray arrows. **(B)** Growth curves and growth parameters of *WT/pIMK2*, *asnB/pIMK2*, and *asnB/pIMK2-asnB* in BHI broth at 30°C (left) and 37°C (right). **(C)** Growth curves and growth parameters of the same strains in BHI broth with 3mMt-CIN at 30°C. **(D)** Growth curves of the *asnB::Himar1* mutant compared to the WT strain in BHI broth with 4mMt-CIN with and without addition of Asn. The growth parameters [lag phase (*λ*), maximum growth rate (μmax), and maximum optical density (ODmax)] represent mean±SD; *n*=3. Values of different strains followed by a common letter are not different at the 5% level of significance.

Several mutants with increased sensitivity to *t*-CIN, an antimicrobial from cinnamon bark essential oil, were previously isolated from a random *Himar1* transposon insertion library of *L. monocytogenes* Scott A (Rogiers et al., 2017). One mutant had the transposon inserted 22bp upstream of the start codon of *asnB* (NCBI accession no. and locus tag are CM001159 and LMOSA_25850), which is predicted to encode an asparagine synthetase, and this mutant was designated *5’asnB::Himar1* (Figure 1A). The transcription start site (TSS) of *asnB* has been reported to be located at −47bp relative to the start codon in *L. monocytogenes* EGD-e and at −50bp in *Listeria innocua* (Wurtzel et al., 2012), making it likely that transcription of *asnB* is disrupted in this mutant. The gene downstream of *asnB* encodes a putative rRNA methylase and is encoded on the opposite strand. An S-adenosylmethionine synthetase gene is located upstream of *asnB* and its stop codon is 136bp away from the start codon of *asnB*. Given their position and orientation, the expression of these flanking genes is unlikely to be strongly affected by the transposon. The genome of the *5’asnB::Himar1* mutant was sequenced and no other mutations were identified. To further study the function of the *asnB* gene, a genetically complemented mutant strain was constructed by chromosomal integration of a pIMK2 plasmid containing an intact *asnB* copy under control of a constitutive promotor (strain *asnB/pIMK2-asnB*), and the WT and *asnB* mutant strain were equipped with an empty pIMK2 vector at the same chromosomal insertion site. More detailed analysis of the *t*-CIN sensitivity indicated that the mutant displayed an extended lag phase (56 vs. 23h for the WT strain) but not a reduced exponential growth rate or stationary phase level (Figure 1C). Furthermore, *t*-CIN tolerance was restored to WT level (20-h lag phase) by genetic complementation (Figure 1C), but not by supplementation of the growth medium with Asn (Figure 1D), suggesting that *t*-CIN sensitivity of the *asnB* mutant is not related to an Asn deficiency that could be the result of impaired Asn synthetase activity.

**Figure 2:**
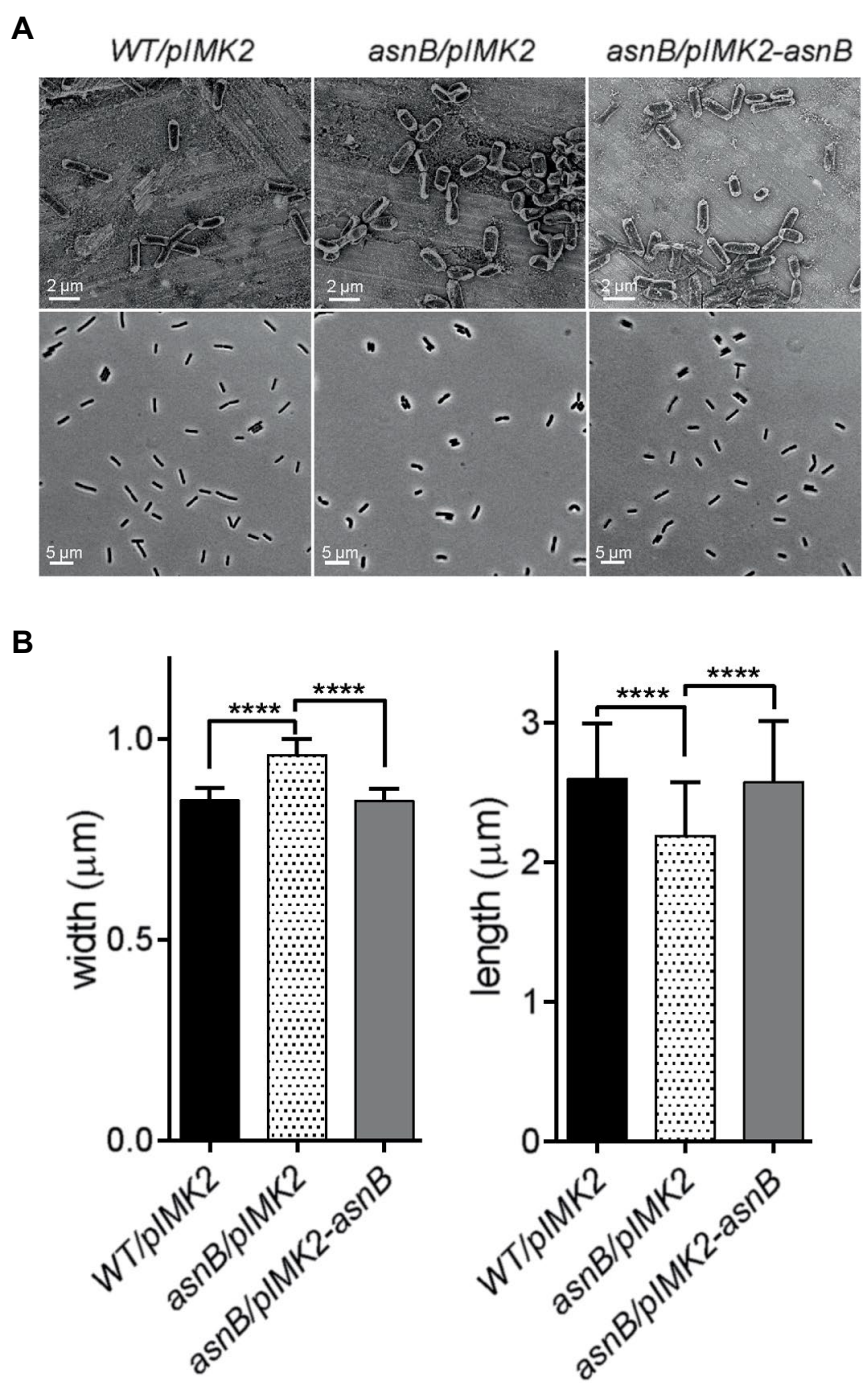
Analysis of cell morphology of the *L. monocytogenes asnB* mutant grown to exponential phase (OD_600_=1) at 30°C. **(A)** SEM (top row) and phase contrast light microscopy images (bottom row) of *WT/pIMK2*, *asnB/pIMK2*, and *asnB/pIMK2-asnB* strains. **(B)** Analysis of cell dimensions of the same strains from phase contrast images using MicrobeTracker software (Sliusarenko et al., 2011). The data represent mean±SD of cell length and width; *n*=300. ****, *p*<0.0001 by two-tailed student’s *t*-test.

Although the *asnB* mutant showed normal growth in BHI broth without *t*-CIN at 30°C, it grew at a lower rate and to a lower stationary phase level at 37°C (Figure 1B). Furthermore, both SEM and optical microscopy observations of exponential and stationary phase cells grown at 30°C revealed that the *asnB* mutant produced “fat rods” that were shorter and thicker than WT cells, and also the presence of some club-shaped cells (Figure 2). Both the growth at 37°C and cell morphology were largely restored by genetic complementation. The observed defects in growth and cell morphology are reminiscent of the phenotype of an *asnB* mutant of *B. subtilis* (Yoshida et al., 1999). Interestingly, *B. subtilis* has two paralogs of *asnB*, designated *asnH* and *asnO*. However, while the three paralogs could restore Asn prototrophy to an *E. coli asnB* mutant, none was essential for producing Asn in *B. subtilis*, and *asnB* was later shown to mediate amidation of the ε-carboxyl group of *m*DAP residues in the peptidoglycan peptide stem (Dajkovic et al., 2017). The role of *asnB* homologs in *m*DAP amidation has also been demonstrated in other Gram-positive bacteria, including *L. plantarum* (Bernard et al., 2011) and *C. difficile* (Ammam et al., 2020). However, unlike these bacteria, which have two or three *asnB* paralogs, *L. monocytogenes* contains only one. This unique constellation triggered us to further study the function(s) of *asnB* in *L. monocytogenes*, including its possible role in virulence, since such a role had not been previously reported in any pathogen.

### The *asnB* Mutant Is Flagella-Less, Defective in Biofilm Formation, and Exhibits Increased Sensitivity to Lysozyme

**Figure 3:**
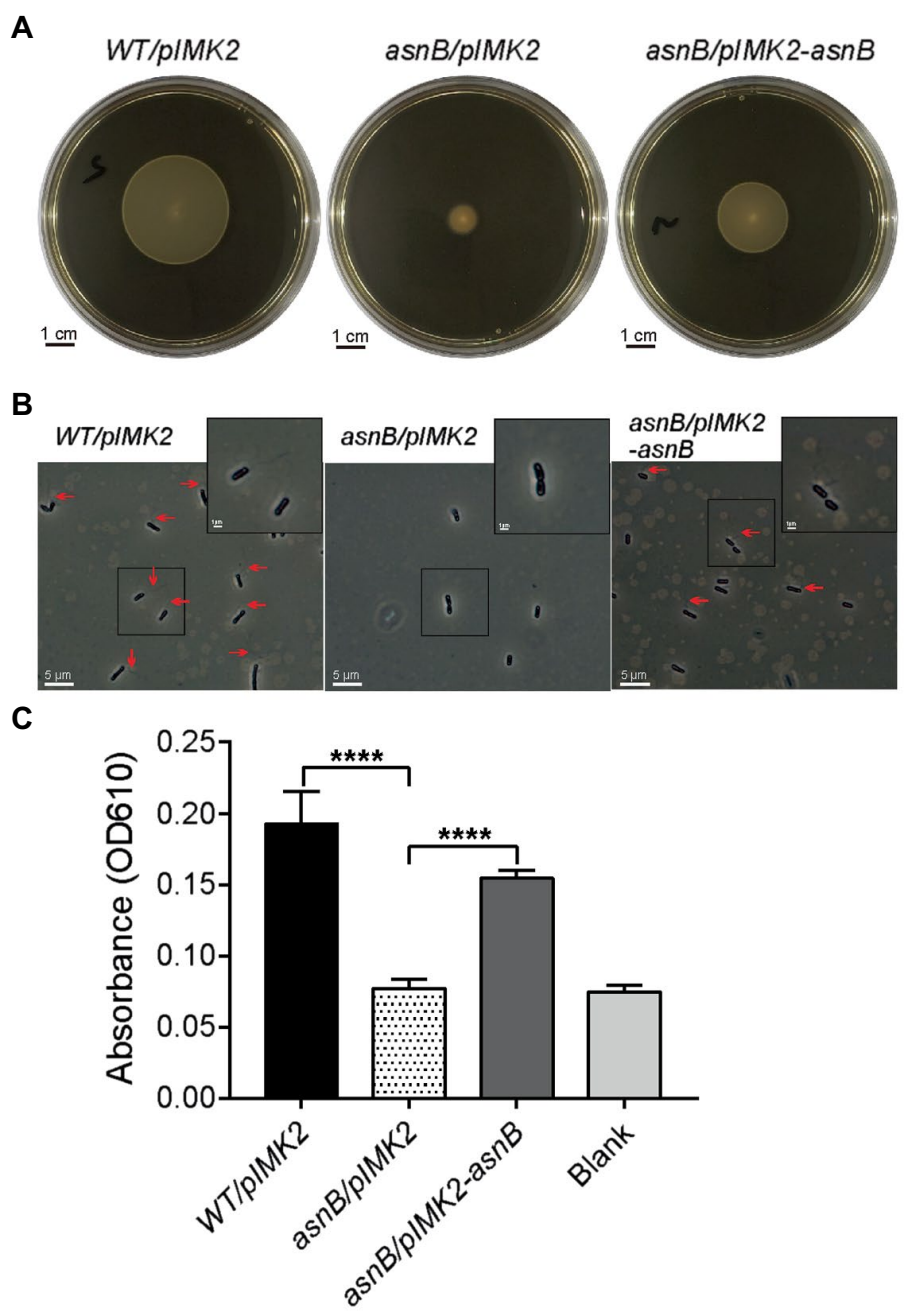
Flagellation, motility, and biofilm formation of the *L. monocytogenes asnB* mutant. **(A)** Swimming motility at 30°C of *WT/pIMK2*, *asnB/pIMK2*, and *asnB/pIMK2-asnB* stabbed in 0.2% BHI agar. Images are representative of three independent tests. **(B)** Detection of flagella by flagellar staining and phase contrast microscopy, in the same strains. For each strain, more than 300 cells from three independent analyses were observed, and flagella were never seen in the *asnB* mutant. **(C)** Biofilm-forming capacity of the same strains in a 96-well polystyrene microplate determined by crystal violet staining. The data represent mean±SD; *n*=4; ****, *p*<0.0001 by two-tailed student’s *t*-test.

In view of the observed cell shape defects of the *asnB* mutant and the possible role of AsnB in *m*DAP amidation, we analyzed some additional phenotypes that could be affected by cell wall perturbation. Swimming motility in 0.2% BHI agar at 30°C was completely lost for the *asnB* mutant, and flagellar staining indicated that this was due to the complete absence of flagella (Figures 3A,B). Motility and flagella production were almost fully restored by genetic complementation. Since flagellar motility was previously shown to play an essential role in *L. monocytogenes* biofilm formation (Lemon et al., 2007), we subsequently determined the biofilm-forming capacity of the *asnB* mutant using the crystal violet staining method. The data showed a complete loss of biofilm-forming capacity of the *asnB* mutant (Figure 3C).

**Figure 4:**
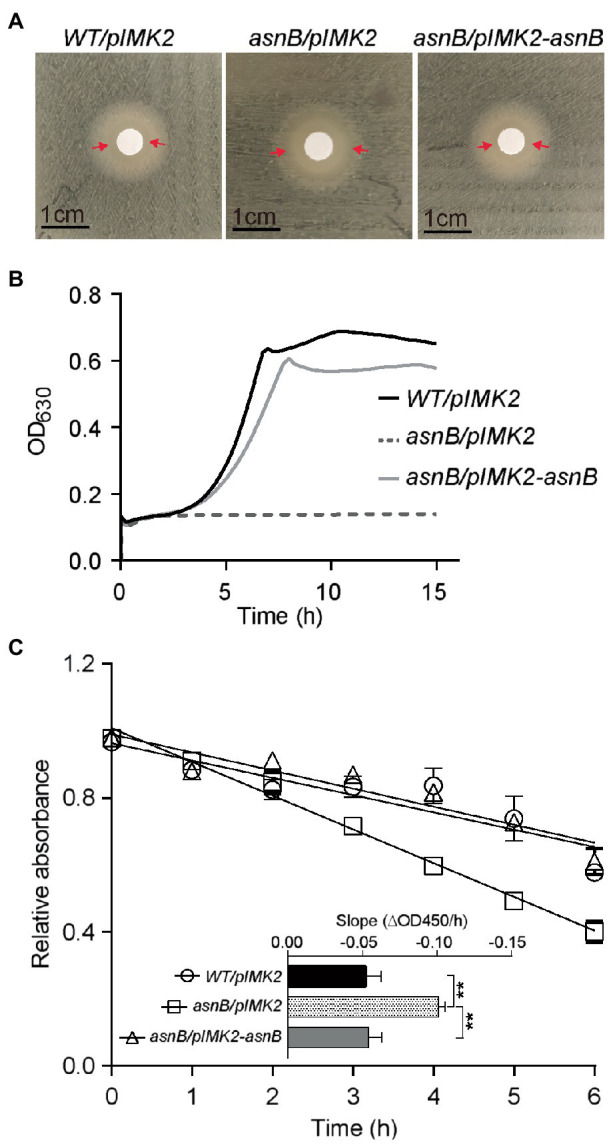
Lysozyme sensitivity of the *L. monocytogenes asnB* mutant. **(A)** Disk diffusion assay of *WT/pIMK2*, *asnB/pIMK2*, and *asnB/pIMK2-asnB* strains. The zone of clearance is indicated with red arrows. Images are representative of at least three independent experiments. **(B)** Growth of the same strains in BHI broth with 1mg/ml lysozyme at 30°C. Graphs are means of three independent experiments. **(C)** Lysis of cell suspensions of the same strains in potassium phosphate buffer in the presence of 0.1μg/ml lysozyme. The relative absorbance on the *y*-axis represents the ratio of absorbance (OD_450_) in the presence of lysozyme vs. water as a control. The inset shows the slopes of linear regression curves from the main graph. Data represent mean±SD; *n*=5; **, *p*<0.0021 by two-tailed student’s *t*-test.

Finally, we investigated the sensitivity of the *asnB* mutant to lysozyme, since *m*DAP amidation had been linked to lysozyme resistance in some bacteria (Levefaudes et al., 2015; Dajkovic et al., 2017). Both the disk diffusion assay and the broth growth inhibition assay revealed lysozyme sensitivity of the *asnB* mutant during growth, while the WT was fully resistant (Figures 4A,B). Furthermore, the *asnB* mutant was also more sensitive to lysozyme in a lysis assay with non-growing cells suspended in a buffer (Figure 4C). Genetic complementation restored WT, or almost WT, lysozyme tolerance in all these assays.

### AsnB Mediates Amidation of *Meso*-Diaminopimelic Acid Residues in Peptidoglycan

**Figure 5:**
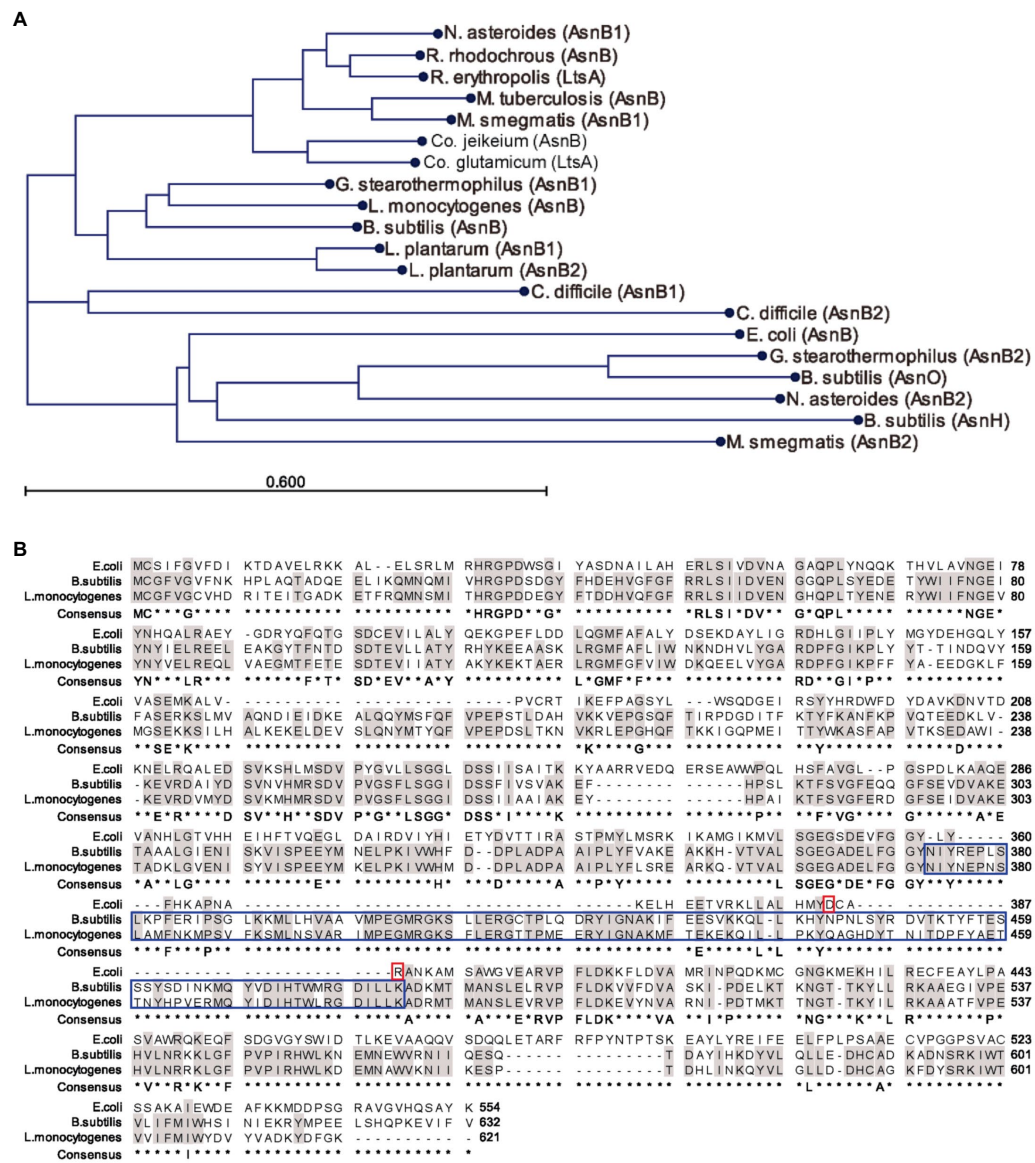
Sequence comparison of AsnB proteins from selected bacteria. **(A)** Phylogenetic tree of AsnB homologs from several Gram-positive bacteria and from *Escherichia coli*, all of which contain *m*DAP in their PG. Bacterial genera: B, *Bacillus*; C, *Clostridioides*; Co, *Corynebacterium*; E, *Escherichia*; G, *Geobacillus*; L, *Lactobacillus*; M, *Mycobacterium*; N, *Nocardia*; and R, *Rhodococcus*. **(B)** Sequence alignment of AsnB from *L. monocytogenes*, *Bacillus subtilis*, and *E. coli*. Identical residues between the three proteins and between AsnB from *B. subtilis* and *L. monocytogenes* are highlighted in gray color. Conserved Asp-binding residues (Ahn, 2013) in AsnB of *E. coli* are highlighted with a red box. The extra amino acid loop characteristic of *m*DAP-amidating AsnB homologs is highlighted with a blue box.

The aberrant cell shape, absence of flagellation, and lysozyme sensitivity suggested a role in *m*DAP amidation rather than in Asn biosynthesis for AsnB in *L. monocytogenes*. To further investigate this possible function, the AsnB amino acid sequence was compared with homologs in other Gram-positive bacteria (Figure 5A). The analysis revealed three major clades, of which one contains no representatives with proven *m*DAP amidation activity. Among the members of this clade are AsnH and AsnO from *B. subtilis*, but also homologs from *Actinobacteria* and AsnB from *E. coli*, which was included for comparison because it is known to be an Asn-producing enzyme. The other two clades, which are more closely related to each other than to the first clade, contain proteins from *Actinobacteria* and *Firmicutes*, respectively, and both contain representatives with demonstrated *m*DAP amidation activity. With 68% sequence identity, *L. monocytogenes* AsnB is most closely related to AsnB from *B. subtilis* (Figure 5B), which was recently shown to mediate PG *m*DAP amidation (Dajkovic et al., 2017). Another highly similar homolog is AsnB1 of *Geobacillus stearothermophilus*, whose *m*DAP in PG is amidated as well (Linnett and Strominger, 1974). In the same clade, AsnB1 of *L. plantarum* was also demonstrated to mediate *m*DAP amidation (Bernard et al., 2011). The same holds for AsnB1 from *C. difficile* (Ammam et al., 2020), but this sequence, together with that of its AsnB2 paralog, forms a small distinct clade.

**Figure 6:**
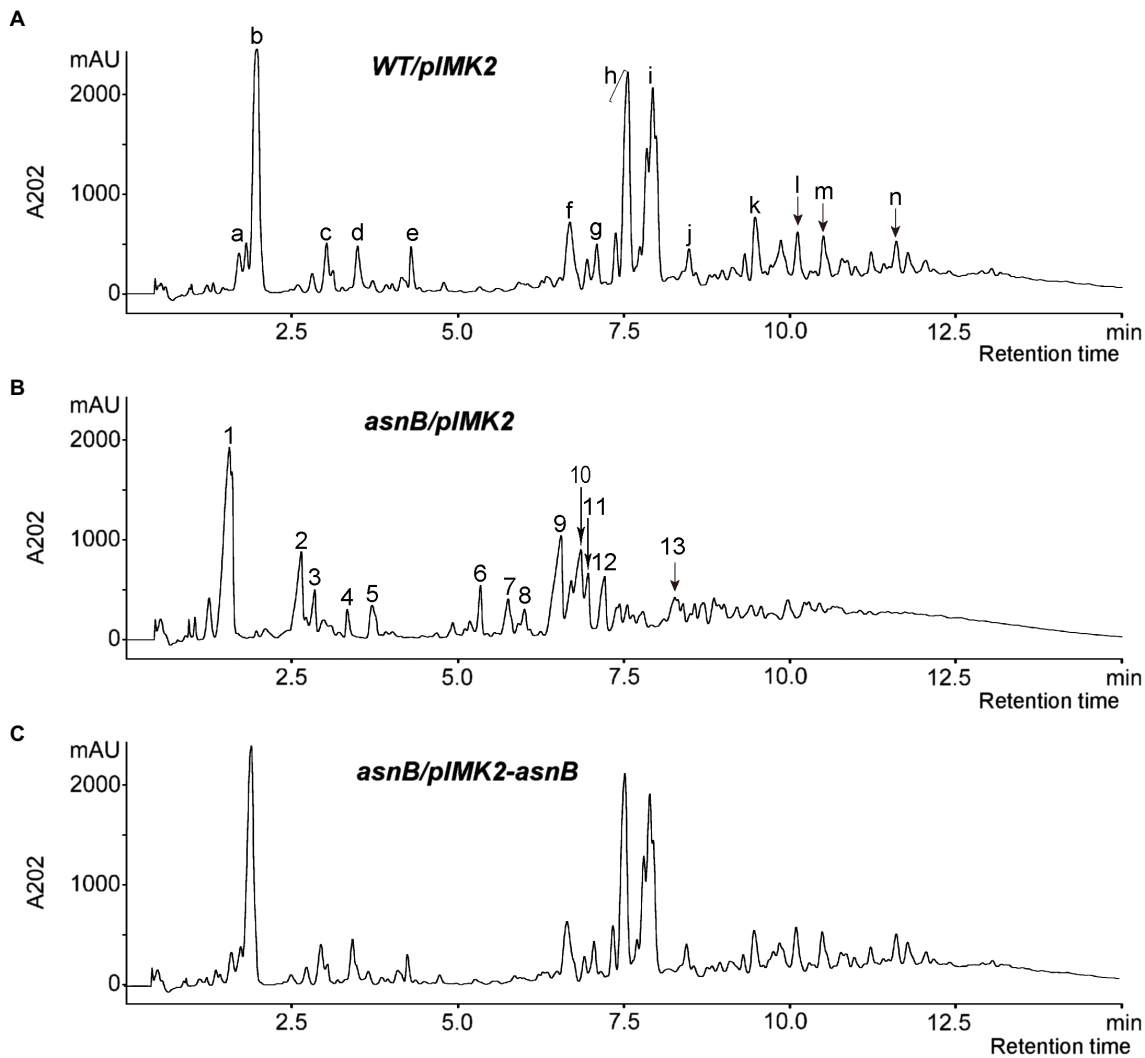
RP-HPLC chromatograms of muropeptides generated by mutanolysin hydrolysis of PG from **(A)** *WT/pIMK2*, **(B)** *asnB/pIMK2*, and **(C)** *asnB/pIMK2-asnB*. The major muropeptide peaks of the chromatogram are annotated with letters and numbers for *WT/pIMK2* and *asnB/pIMK2*, respectively. The mass and identity of the labeled peaks are listed in Supplementary Table S2. Disaccharide tripeptides identified in peak b of *WT/pIMK2* and peak 1 of *asnB/pIMK2* were further analyzed by MS–MS (Supplementary Figure S1).

To provide direct evidence for the involvement of AsnB in *m*DAP amidation, the PG structure of the *WT/pIMK2*, *asnB/pIMK2*, and *asnB/pIMK2-asnB* strains was analyzed by enzymatic hydrolysis followed by RP-HPLC and mass spectrometry. The muropeptide profiles of *WT/pIMK2* and *asnB/pIMK2-asnB* by RP-HPLC were almost indistinguishable (Figure 6). In contrast, the profile of *asnB/pIMK2* was strikingly different with the major peaks shifted toward lower retention times.

MALDI-TOF MS analysis was conducted to identify the muropeptides corresponding to peaks in the chromatograms (Supplementary Table S2). MS analysis indicated that part of GlcNAc residues was deacetylated, and part of MurNAc residues was O-acetylated, which is consistent with previous studies (Boneca et al., 2007; Aubry et al., 2011; Burke et al., 2014). The dimer peaks in the muropeptide profile of *asnB/pIMK2* (Figure 6B, peaks 7–13) are relatively smaller than those of *WT/pIMK2* (Figure 6A, peaks f to k), indicating a reduced ratio of dimers to monomers, thus suggesting a reduced cross-linking of PG. In addition, the results indicated that most stem peptides in *WT/pIMK2* muropeptides bear one amidation. In contrast, no amidation was detected in the *asnB/pIMK2* mutant strain. Since amidation can not only occur on the ε-carboxyl group of *m*DAP, but also on the α-carboxyl groups of D-Glu, tandem mass spectrometry (MS/MS) was performed on the major monomer muropeptide (disaccharide tripeptide), which corresponds to peak b and peak 1 in the chromatogram of *WT/pIMK2* and *asnB/pIMK2*, respectively (Supplementary Figure S1). This confirmed that only *m*DAP in the PG stem peptide is amidated in *WT/pIMK2*, whereas it is not in *asnB/pIMK2* mutant. Altogether, these results provide convincing evidence that *L. monocytogenes* AsnB is an amidotransferase that amidates *m*DAP residues in the PG stem peptide.

### *m*DAP Amidation Is Required for Host Cell Invasion

**Figure 7:**
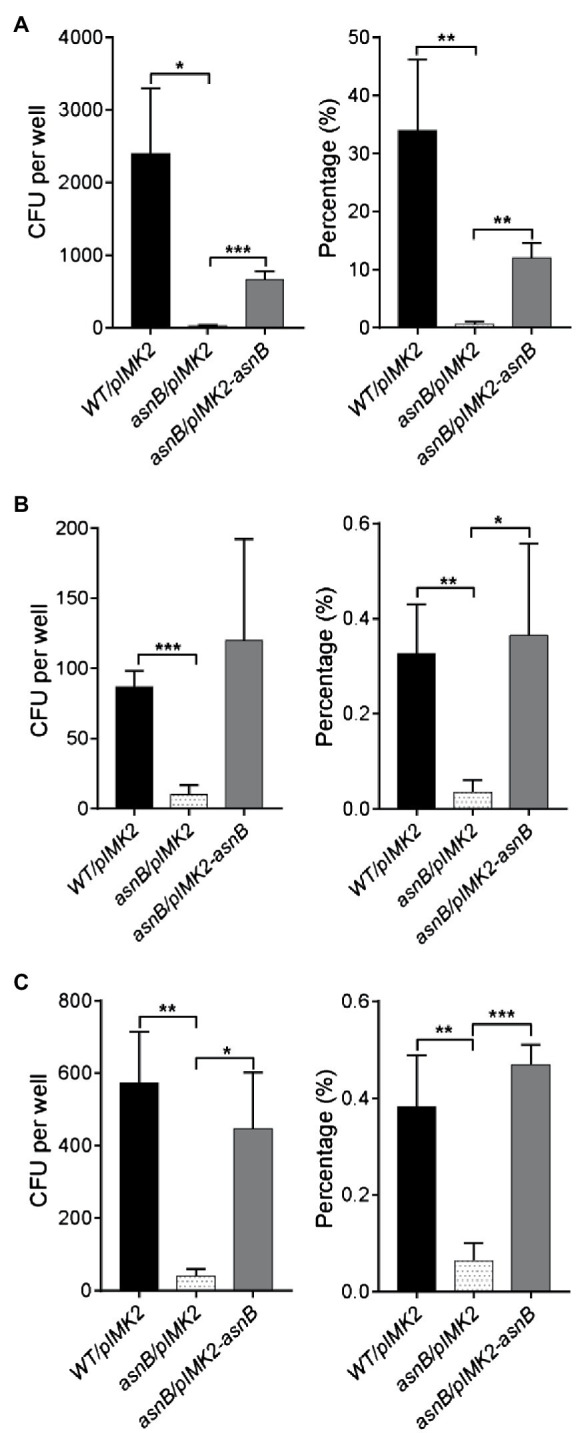
Analysis of cell invasion by the *L. monocytogenes asnB* mutant using gentamicin invasion assays. The number of intracellular bacteria was enumerated by CFU counts (left panel) and the invasion efficiency was expressed as the percentage of intracellular bacteria relative to the bacterial inoculum (right panel). **(A)** Human JEG-3 placental cells were infected with *WT/pIMK2*, *asnB/pIMK2*, or *asnB/pIMK2-asnB* bacteria for 4h (1h to allow bacterial entry and 3h of treatment with gentamicin, which kills extracellular bacteria). **(B,C)** Human Caco-2 intestinal cells were infected with *WT/pIMK2*, *asnB/pIMK2*, or *asnB/pIMK2-asnB* bacteria at either exponential **(B)** or stationary **(C)** phase for 1h (0.5h to allow bacterial entry and 0.5h of treatment with gentamicin). All data represent mean±SD; *n*=3; *, *p*<0.05; **, *p*<0.01; ***, and *p*<0.001 by two-tailed student’s *t*-test.

Peptidoglycan N-deacetylation and O-acetylation are critical for virulence of *L. monocytogenes* by mediating escape of the immune response (Boneca et al., 2007; Aubry et al., 2011; Rae et al., 2011). However, the possible role of *m*DAP amidation in virulence has not yet been studied in *L. monocytogenes*, nor in any other pathogen, and was therefore addressed here by using a gentamicin cell invasion assay. Firstly, human JEG3 placental cells were infected with stationary phase bacteria for 1h, followed by incubation with gentamicin for an additional 3h, and the invasion efficiency was expressed as the ratio of the number of recovered bacteria to the number of bacteria initially applied. The results highlighted an important defect in cellular invasion of the *asnB/pIMK2* mutant, with a reduction in invasion efficiency to less than 2% of the level of the WT strain, while complementation resulted in a restoration of invasion efficiency to about 30% of that of the WT strain (Figure 7A). To determine whether the attenuation of cell invasion caused by *asnB* inactivation also occurred in another cell type, we next performed invasion assays in human Caco-2 intestinal cells. The assay was modified to test bacteria in either stationary or exponential phase and to shorten the duration of the infection, in order to avoid the intracellular replication of bacteria, which might be impacted by AsnB deficiency. Caco-2 cells were thus exposed to bacteria for only 1h, of which 30min was allocated for entry of bacteria into the cells, and another 30min to the elimination of the extracellular bacteria with gentamicin. Entry efficiency was expressed as the ratio of bacteria recovered after 1h of infection to the initial number of bacteria applied (Figures 7B,C). Compared to the WT strain, the entry efficiency of the *asnB/pIMK2* mutant at exponential and stationary phase was 10 and six times lower, respectively (Figures 7B,C). Furthermore, the entry efficiency was fully restored by complementation. Taken together, these results indicate that *asnB* inactivation impairs *L. monocytogenes* entry into epithelial cells.

### Loss of *m*DAP Amidation Reduces Anchoring of the Invasion Protein InlA to the Cell Surface

**Figure 8:**
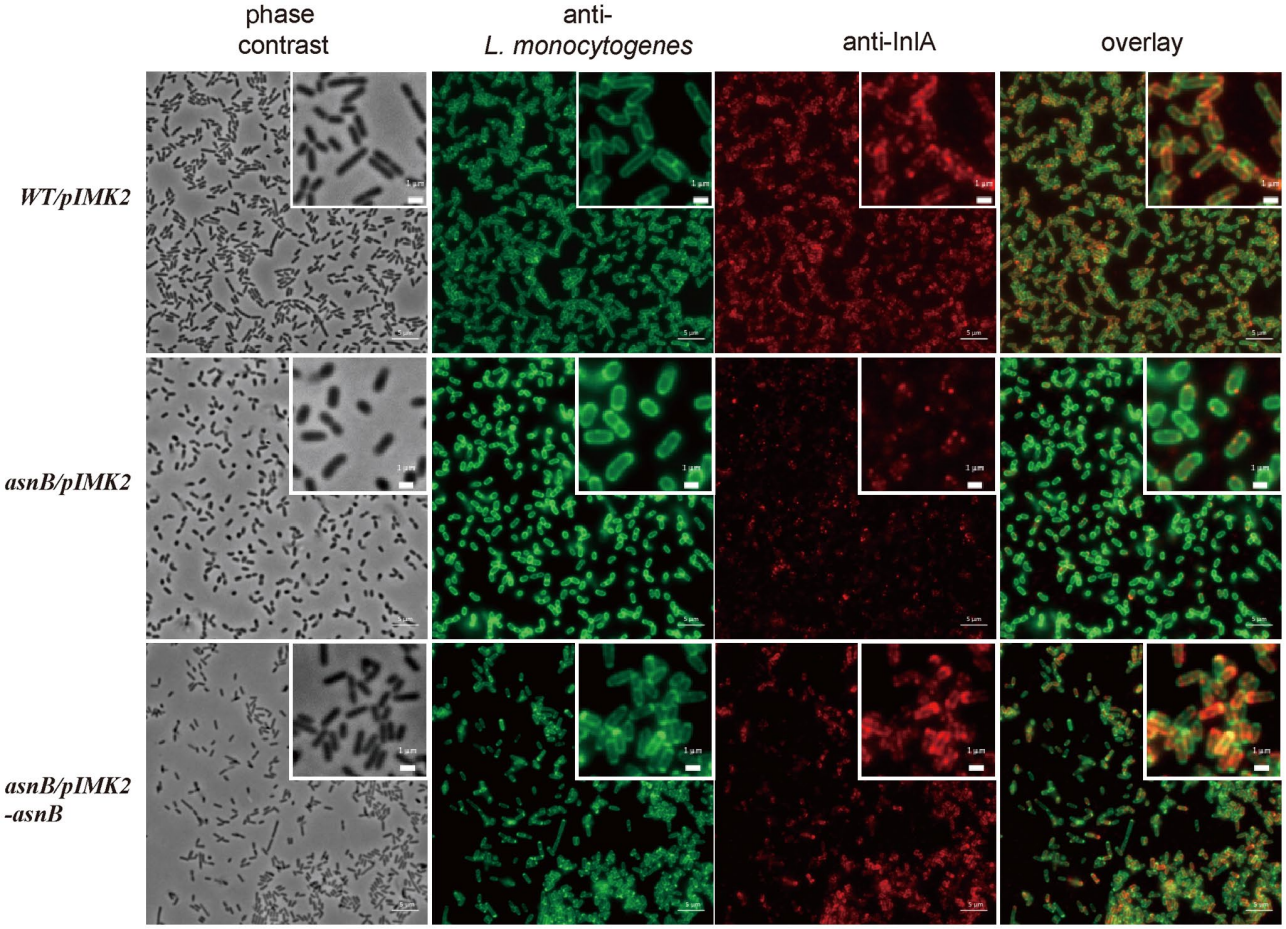
Immunofluorescence microscopy analysis of the invasion protein InlA on the surface of *L. monocytogenes WT/pIMK2*, *asnB/pIMK2*, and *asnB/pIMK2-asnB* strains. Bacteria were visualized using phase contrast microscopy (first lane) and with immunofluorescence staining with a polyclonal anti-*L. monocytogenes* antibody (second lane) and a mixture of two monoclonal anti-InlA antibodies (third lane). Bacteria (in green) and InlA (in red) are shown together in an overlay composition (last lane). Scale bar 5μM. A higher magnification is shown on the upper right corner. Scale bar 1μM.

Internalins are a family of leucine-rich repeat proteins that play an important role in the *Listeria* infection process (Bierne et al., 2007). The majority of internalins is LPXTG proteins covalently bound to the bacterial cell wall by the Sortase A (SrtA) enzyme, the best characterized being InlA, which mediates the adhesion and internalization of *L. monocytogenes* into epithelial cells (Gaillard et al., 1991; Mengaud et al., 1996). Interestingly, InlA is anchored to the bacterial surface by covalent linkage to *m*DAP in the peptidoglycan stem peptide (Dhar et al., 2000). Therefore, and because the *asnB* mutant has a defect in cell entry, we tested whether the *asnB* mutant is defective in cell wall anchoring of InlA. The presence of InlA on the bacterial surface was analyzed by immunofluorescence using InlA-specific monoclonal antibodies, as previously described for the study of a sortase A mutant (Bierne et al., 2002). In comparison with the WT strain, detection of InlA on the surface of the *asnB/pIMK2* mutant cells was considerably reduced (Figure 8). In contrast, InlA was clearly detected on the surface of the complemented strain, indicating that expression of *asnB* in the mutant restored surface anchoring. Thus, amidation at the ε-carboxyl group of *m*DAP appears to be required for efficient cross-linking of InlA to *m*DAP.

## Discussion

In this work, we demonstrate that the *asnB* gene of *L. monocytogenes* is required for amidation of the ε-carboxyl group of *m*DAP in the peptidoglycan stem peptide (Figure 6; Supplementary Table S2). Based on amino acid sequence similarity, the predicted gene product AsnB belongs to a family of Gln-hydrolyzing amidotransferases designated as asparagine synthases (E.C. 6.3.5.4). While AsnB proteins are indeed involved in Asn synthesis in several bacteria, representatives in an increasing number of Gram-positive bacteria, including *L. plantarum* (Bernard et al., 2011), *C. glutamicum* (Levefaudes et al., 2015), *B. subtilis* (Dajkovic et al., 2017), and *C. difficile* (Ammam et al., 2020), have been shown to catalyze amidotransfer from Gln to the ε-carboxyl group of *m*DAP in the stem peptide of bacterial peptidoglycan rather than to Asp. Although the role of AsnB had not been studied in *L. monocytogenes*, there were at least two reasons to anticipate that it would also have *m*DAP modifying activity. Firstly, structural analysis had already revealed *m*DAP residues to be amidated in two *L. monocytogenes* serotype 1/2a strains (Boneca et al., 2007; Burke et al., 2014). Secondly, *L. monocytogenes* AsnB shares most sequence similarity to AsnB from *B. subtilis* and other homologs known to amidate *m*DAP (Figure 5). Furthermore, *L. monocytogenes* AsnB lacks the conserved Asp-binding residues (D and R) of enzymes with Asn synthesizing activity, and it has the extra loop in the substrate binding region that is characteristic of the *m*DAP-amidating enzymes (Ahn, 2013). We did not analyze whether AsnB from *L. monocytogenes* can restore Asn auxotrophy in an *E. coli asnB* mutant and thus cannot exclude that it is a promiscuous enzyme similar to its *B. subtilis* counterpart (Yoshida et al., 1999). However, since neither *t*-CIN tolerance or normal cell morphology was restored in the *asnB* mutant by supplying additional Asn in the growth medium, it seems unlikely that these phenotypes result from Asn deficiency and that AsnB plays a role in Asn synthesis in *L. monocytogenes*. Besides the AsnB-catalyzed amidotransfer from Gln to asp., two alternative Asn biosynthesis routes have been documented in bacteria. One is the direct amidation of Asp with ammonium by an AsnA-type asparagine synthetase. The other one is a tRNA-dependent transamidation, in which a tRNA^Asn^ is first “mischarged” with Asp by a nondiscriminating aspartyl tRNA synthetase (AspS), and Asp is subsequently amidated by a Gln-hydrolyzing asparaginyl-tRNA synthase (GatABC). Based on its genome sequence, it is predicted that *L. monocytogenes* does not produce an AsnA-like enzyme but can synthesize Asn *via* the tRNA-dependent pathway, like many other Gram-positive bacteria.

Like in *B. subtilis*, *L. plantarum*, and *C. glutamicum* (Bernard et al., 2011; Levefaudes et al., 2015; Dajkovic et al., 2017), the loss of AsnB-dependent *m*DAP amidation resulted in altered cell morphology and growth defects in *L. monocytogenes*. Such effects were not seen in *C. difficile*, but this may be because AsnB is expressed only upon induction with vancomycin in this organism (Ammam et al., 2020). Given the key role of peptidoglycan in cell shape determination and the complexity of bacterial cell morphogenesis, it is not surprising that chemical modifications of PG have an impact on cell shape, cell division, and growth. Nevertheless, the mutational elimination of PG O-acetylation by OatA, PG N-deacetylation by PgdA, or both simultaneously did not affect growth and was not reported to affect cell morphology (Rae et al., 2011). One possible explanation for this different impact is that O-acetylation and N-deacetylation do not modify the electrostatic charge of PG in the normal pH range of growth, whereas amidation of the ε-carboxyl group of *m*DAP reduces the number of negatively charged carboxylate groups. Similarly, the amidation of D-Glu in the PG stem peptide that occurs in some bacteria also reduces negative charges on PG and was found to be essential for normal growth in *S. aureus* (Münch et al., 2012). A phenotype of the AsnB mutant that can be directly related to (loss of) PG modification is its lysozyme sensitivity. This phenotype was already reported for *asnB* mutants of *B. subtilis* and *C. glutamicum* (Levefaudes et al., 2015; Dajkovic et al., 2017). Besides N-deacetylation and O-acetylation (Boneca et al., 2007; Rae et al., 2011; Burke et al., 2014), *m*DAP amidation is therefore the third PG modification implicated in lysozyme resistance in *L. monocytogenes*. How the different modifications interact, and their precise contribution and hierarchy in conferring lysozyme resistance are not entirely clear and may vary among strains and environmental conditions. For example, deletion of *oatA* caused lysozyme sensitivity in *L. monocytogenes* strain EGD-e (Aubry et al., 2011) but not in strain 10403S although it exacerbated lysozyme sensitivity of a *pgdA* mutant in this strain (Rae et al., 2011; Burke et al., 2014). *Listeria monocytogenes* is known to also infect and even cause listeriosis in several other vertebrates, including birds and fish, some of which produce additional or different types of lysozymes with different substrate specificity (Miettinen and Wirtanen, 2005; Hellström et al., 2008; Callewaert and Michiels, 2010), and the presence and ability to modulate the activity of different PG modifying enzymes may be an adaptation to this promiscuous lifestyle.

In several bacterial pathogens, modifications that render PG resistance to lysozyme support the ability to establish infections (Davis and Weiser, 2011), and at least two different mechanisms may explain this. First, PG modification protects the bacteria from the direct action of lysozyme as a first-line antibacterial defense actor of the innate immune system in those parts of the body where it is abundant, such as the blood, saliva, tears, milk, phagocytes, and mucosal surfaces. Additionally, PG modification also suppresses the lysozyme-mediated release of PG fragments that modulate the host immune response upon binding to a range of pattern recognition receptors. Among these, the cytosolic receptor NOD1 is the major receptor of PG fragments containing the D-Glu-*m*DAP stem peptide residues (Chamaillard et al., 2003), but amidation of *m*DAP significantly blocks its recognition of PG fragments (Girardin et al., 2003; Vijayrajratnam et al., 2016). The response of the NOD2 receptor, on the other hand, is weak for fragments containing non-amidated *m*DAP but considerably enhanced by *m*DAP amidation (Girardin et al., 2003). Therefore, *m*DAP amidation is expected to modulate the immune response to *L. monocytogenes*, potentially leading to a more efficient clearing of AsnB-deficient mutants by the immune system.

**Figure 9:**
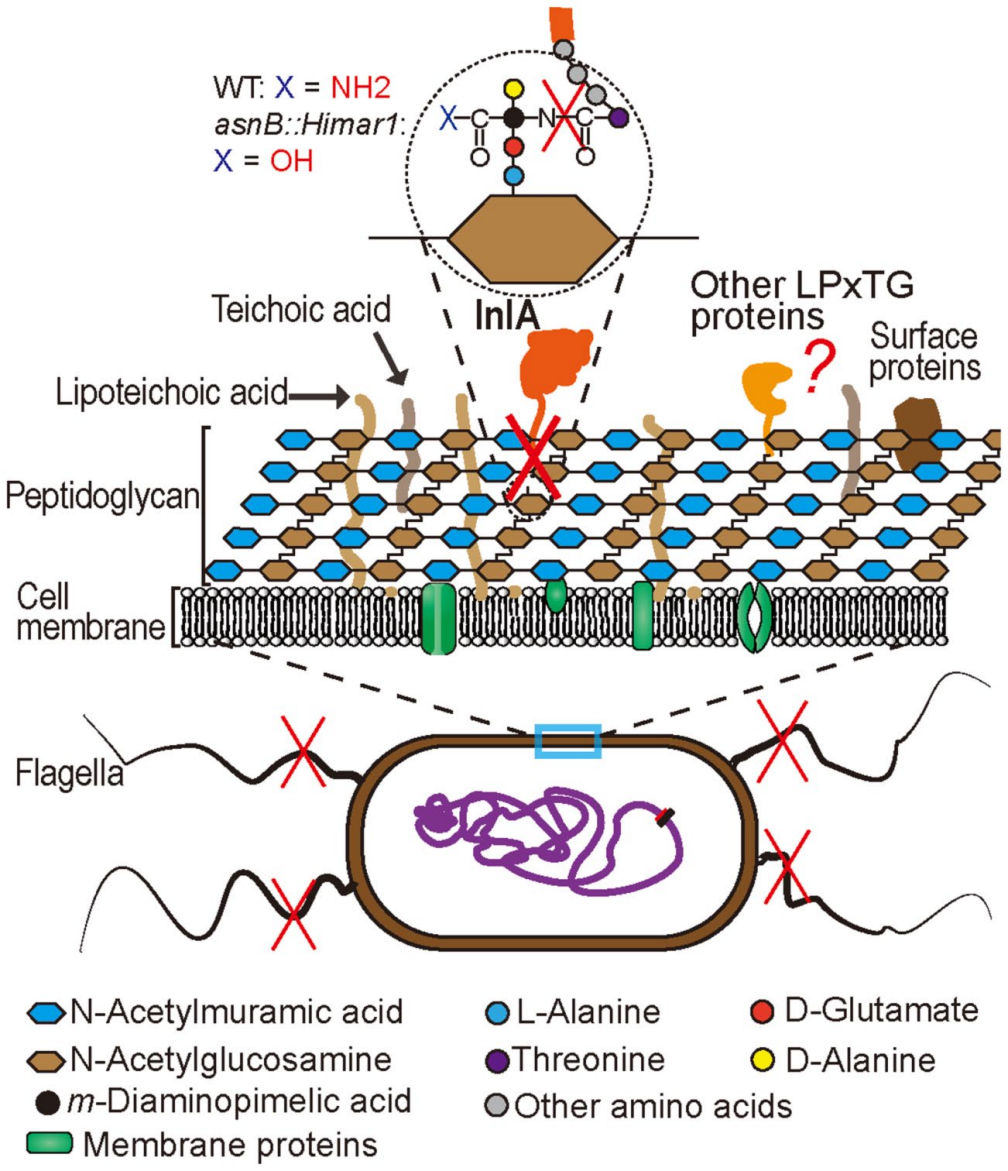
Schematic representation of cell surface changes resulting from AsnB deficiency in *L. monocytogenes*. The direct effect of loss of AsnB activity is the absence of amidation of the *m*DAP ε-carboxyl group (shown in inset in top of the figure). Indirect consequences include inefficient InlA display at the cell surface, probably because it can no longer be cross-linked to the ε-amino group of *m*DAP and the loss of flagella (indicated by red cross over the structure). Other surface proteins which, like InlA, are exported and cross-linked *via* an LPXTG motif may also fail to be anchored to the cell surface (red question mark).

In the present work, we found that AsnB is required for *Listeria* invasion of epithelial cells, which is mainly mediated by the bacterial surface protein InlA through its interaction with the host receptor E-cadherin (Gaillard et al., 1991; Mengaud et al., 1996). InlA plays a critical role in the ability of *L. monocytogenes* to cross epithelial barriers, such as the intestinal and placental barriers (Lecuit, 2005). Here, we showed a reduced presence of InlA on the surface of the *asnB* mutant, which can explain the invasion deficiency in human intestinal Caco-2 cells and placental JEG-3 cells. InlA contains both an N-terminal signal peptide and a C-terminal LPXTG sorting signal (Dhar et al., 2000). After translation, InlA is secreted across the bacterial membrane *via* the Sec secretory pathway, which recognizes and cleaves off the N-terminal signal peptide of the protein (Bierne and Dramsi, 2012). Subsequently, SrtA (Sortase A) cleaves the protein after the threonine residue of the LPXTG motif in its C-terminal part and covalently links the protein to the PG lipid intermediate II, by forming a new peptide bond between the carboxyl group of Thr and the free ε-amino group of *m*DAP (Dhar et al., 2000; Bierne et al., 2002). The lipid-linked protein is subsequently incorporated into the mature cell wall through the transglycosylation and transpeptidation reactions (Bierne and Dramsi, 2012). Although the ε-amino group of *m*DAP remains available, our results indicate that deamidation of the ε-carboxyl group may prevent the cross-linking of InlA. Several possible reasons for this can be conceived. First, the increased negative charge of deamidated *m*DAP may reduce the binding affinity or even cause repulsion between the SrtA-InlA complex and PG precursor lipid II. Second, deamidation may induce a conformational change that prevents the correct positioning of the SrtA-InlA complex and the PG precursor lipid II (Dhar et al., 2000). It should be noted that besides InlA, *L. monocytogenes* produces many other LPXTG motif containing proteins (40 in strain EGD-e), several of which have been implicated in virulence as well (Bierne and Cossart, 2007; Bierne and Dramsi, 2012; Mariscotti et al., 2012), and the binding of these proteins to a non-amidated PG may also be affected (Figure 9). The literature is not consistent about whether InlA and other LPXTG proteins are linked to amidated or non-amidated *m*DAP in *L. monocytogenes*. To our knowledge, only one study has investigated the anchor structure of this type of proteins to the cell wall in *L. monocytogenes*, and it depicts InlA linked to non-amidated *m*DAP (Dhar et al., 2000). Remarkably, the authors also propose an amidated iGlu (i.e., an iGln) residue in the structure of the stem peptide. According to our MS/MS results (see Supplementary Figure S1 of our paper), there is no ambiguity to say that *m*DAP is amidated, whereas iGlu is not. Notably, this is consistent with the presence in the *L. monocytogenes* genome of an *m*DAP-amidating *asnB* gene (this work), and with the absence of *murT/gatD* genes encoding the enzyme complex catalyzing iGlu amidation in other bacteria (data not shown). Probably the MS data presented by Dhar et al. (2000) were obtained with mass spectrometers that were much less accurate than today’s ones and thus amidation of *m*DAP or iGlu could not be clearly assigned, since amidation results in a loss of only 1Da of mass.

AsnB deficiency was also accompanied by a loss of bacterial motility due to the inability to produce flagella in *L. monocytogenes* (Figures 3, 9). Remarkably, this phenotype has not been reported previously in *asnB* mutants of other bacteria including the closely related *B. subtilis*. The flagellar apparatus is embedded in the bacterial cell wall, where it interacts directly with the PG layer *via* several components of its basal body (Macnab, 2003). Its assembly is a complex and highly coordinated process that requires local and controlled PG hydrolase activity to mediate passage of the flagellar rod through the PG layer (Macnab, 2003). As such, it is easy to envision that the increased negative charge of the non-amidated PG, or the absence of one or more PG-linked proteins, may compromise flagellar assembly. However, the precise reason for the lack of flagella remains to be elucidated. The lack of flagella and flagellum-mediated motility may also explain the reduced biofilm formation of the *asnB* mutant (Lemon et al., 2007), although other changes in surface properties may also contribute.

In conclusion, this work demonstrates that AsnB of *L. monocytogenes* is a Gln-hydrolyzing amidotransferase that amidates *m*DAP residues in the stem peptide of *L. monocytogenes* PG. Loss of AsnB does not affect growth in BHI broth at 30°C but causes sensitivity to *t*-cinnamaldehyde and lysozyme, reduced epithelial cell invasion and thus probably reduced virulence, loss of flagella and motility, and reduced biofilm formation. These results indicate the importance of PG amidation both for host infection and for life in the non-host environment.

## Data Availability Statement

The raw data supporting the conclusions of this article will be made available by the authors, without undue reservation.

## Author Contributions

LS and CM: conceptualization. LS and GR: investigation. LS: writing – original draft preparation. LS, CM, M-PC-C, and HB: writing – review and editing. CM: supervision and administration. PC and M-PC-C: bacterial peptidoglycan structure analysis. HB: bacteria invasion assays and immunostaining. All authors contributed to the article and approved the submitted version.

## Funding

This work was funded by research grants from the Research Foundation-Flanders (FWO) (G.0C77.14N), the KU Leuven Research Fund (METH/14/03), and the French National Research Agency (ANR PERMALI) (# ANR-20-CE35-0001-01).

## Conflict of Interest

The authors declare that the research was conducted in the absence of any commercial or financial relationships that could be construed as a potential conflict of interest.

## Publisher’s Note

All claims expressed in this article are solely those of the authors and do not necessarily represent those of their affiliated organizations, or those of the publisher, the editors and the reviewers. Any product that may be evaluated in this article, or claim that may be made by its manufacturer, is not guaranteed or endorsed by the publisher.
