# Supplementary material for: AsnB Mediates Amidation of Meso-Diaminopimelic Acid Residues in the Peptidoglycan of Listeria monocytogenes and Affects Bacterial Surface Properties and Host Cell Invasion
Source: Front Microbiol. 2021 Oct 15;12:760253. doi: 10.3389/fmicb.2021.760253 (PMC8554201; doi:10.3389/fmicb.2021.760253)
Supplement: Supplementary file 1 [file Data_Sheet_1.docx]

Supplementary Material

# Supplementary Tables

Table S1. List of protein sequences used in the phylogeny tree construction (Fig. 5).

| GenBank acc. no. | Organism | Protein name | Length, а.a |
| --- | --- | --- | --- |
| WP_019046170.1 | *Nocardia asteroides* | AsnB1 | 647 |
| WP_022565516.1 | *Nocardia asteroides* | AsnB2 | 613 |
| P22106 | *Escherichia coli* | AsnB | 554 |
| WP_053414543.1 | *Geobacillus stearothermophilus* | AsnB1 | 635 |
| WP_013523091.1 | *Geobacillus stearothermophilus* | AsnB2 | 615 |
| WP_004398625 | *Bacillus subtilis 168* | AsnB | 632 |
| WP_003233080 | *Bacillus subtilis 168* | AsnO | 614 |
| WP_003244103 | *Bacillus subtilis 168* | AsnH | 747 |
| WP_011729689 | *Mycolicibacterium smegmatis* | AsnB1 | 658 |
| WP_011728459 | *Mycolicibacterium smegmatis* | AsnB2 | 601 |
| WP_003436426 | *Clostridioides difficile 630* | AsnB1 | 624 |
| WP_011860775 | *Clostridioides difficile 630* | AsnB2 | 527 |
| WP_057717704.1 | *Lactobacillus plantarum* | AsnB2 | 633 |
| WP_054399562.1 | *Lactobacillus plantarum* | AsnB1 | 633 |
| WP_003731601 | *Listeria monocytogenes Scott A* | AsnB | 621 |
| WP_005296050 | *Corynebacterium jeikeium* | AsnB | 645 |
| BAA89484 | *Corynebacterium glutamicum* | LtsA | 640 |
| WP_055372007 | *Mycobacterium tuberculosis* | AsnB | 652 |
| WP_020908107.1 | *Rhodococcus erythropolis* | LtsA | 641 |
| WP_160875722.1 | *Rhodococcus rhodochrous* | AsnB | 641 |

Table S2. Mass spectrometry analysis of major muropeptide peaks in the chromatogram of *WT/pIMK2* and *asnB/pIMK2* strain (Fig. 6). The deduced muropeptide structures and their corresponding m/z values are shown in the Table. The presence of amidation on the muropeptides is indicated with red color.

| ***WT/pIMK2*** | | | | ***asnB/pIMK2*** | | | |
| --- | --- | --- | --- | --- | --- | --- | --- |
| **Peak#*** | **Muropeptide**** | **Observed**  **m/z**  **[M+Na]^+^** | **Calculated m/z**  **[M+Na]^+^** | **Peak*** | **Muropeptide**** | **Observed**  **m/z**  **[M+Na]^+^** | **Calculated m/z**  **[M+Na]^+^** |
|  | **Monomers** |  |  |  | **Monomers** |  |  |
| a | Tri(NH_2_)-Ac | 850.25 | 850.37 | 1 | Tri | 893.19 | 893.36 |
| b | Tri(NH_2_) | 892.21 | 892.38 |  | Tri-Ac | 850.92 | 851.35 |
| c | Di | 720.96 | 721.27 | 2 | Tetra | 964.26 | 964.4 |
|  | Tetra(NH_2_)-Ac | 921.25 | 921.40 | 3 | Tetra-Ac | 922.25 | 922.39 |
| d | Tetra(NH_2_) | 963.30 | 963.41 | 4 | Tri+Ac | 935.25 | 935.37 |
|  | Tetra(NH_2_)-Ac | 921.25 | 921.40 | 5 | Penta | 1035.25 | 1035.43 |
| e | Tri(NH_2_)+Ac | 934.21 | 934.39 | 6 | Tri Anh | 873.12 | 873.33 |
|  | **Dimers** |  |  |  | **Dimers** |  |  |
| f | TriTetra(NH_2_) | 1815.93 | 1815.77 | 7 | TriTetra-Glc | 1613.83 | 1613.68 |
|  | TetraTetra(NH_2_)-GM | 1406.73 | 1406.62 | 8 | TriTetra-Ac-Glc | 1571.82 | 1571.67 |
| g | TriTetra(NH_2_) | 1815.93 | 1815.77 | 9 | TriTetra | 1816.88 | 1816.76 |
|  | ds4a4 | 1406.73 | 1406.62 | 10 | TriTetra-Ac | 1774.86 | 1774.75 |
| h | TriTetra(2 NH_2_) | 1814.91 | 1814.79 | 11 | TriTetra-2Ac | 1732.88 | 1732.74 |
|  | TriTetra(2 NH_2_)-Ac -Glc | 1569.83 | 1569.70 | 12 | TetraTetra | 1887.94 | 1887.79 |
|  | TriTetra(NH_2_)-Ac | 1773.90 | 1773.76 | 13 | TriTetra+Ac | 1858.95 | 1858.77 |
| i | TriTetra(2 NH_2_)-Ac | 1772.95 | 1772.78 |  |  |  |  |
|  | TriTetra(2 NH_2_)-2Ac | 1730.94 | 1730.77 |  |  |  |  |
| j | TetraTetra(2 NH_2_) | 1886.04 | 1885.83 |  |  |  |  |
| k | TetraTetra(2 NH_2_)+Ac | 1856.96 | 1856.80 |  |  |  |  |
|  | **Trimers** |  |  |  |  |  |  |
| l | TriTetraTetra(3 NH_2_) | 2736.94 | 2737.20 |  |  |  |  |
| m | TriTetraTetra(3 NH_2_)-Ac | 2695.01 | 2695.19 |  |  |  |  |
| n | TriTetraTetra(3 NH_2_)+Ac | 2778.96 | 2779.21 |  |  |  |  |
|  | TriTetra(2 NH_2_)Anh-Ac | 1752.89 | 1752.75 |  |  |  |  |

*: Peak # was assigned based on RP-HPLC chromatography (Fig.6).

**: Di, disaccharide dipeptide (L-Ala-D-iGlu); Tri: disaccharide tripeptide (L-Ala-D-iGlu-*m*DAP); Tetra: disaccharide tetrapeptide (L-Ala-D-iGlu-mDAP-D-Ala); Penta: disaccharide pentapeptide (L-Ala-D-iGlu-mDAP-D-Ala-D-Ala); disaccharide, GlcNAc-MurNAc; (x NH_2_): number of amidated residues; (-Ac): deacetylation of GlcNAc; (+Ac): O-acetylation on MurNAc; -Glc: loss of 1 GlcNAc; -GM: loss of disaccharide GlcNAc-MurNAc; Anh: anhydroMurNAc.

##
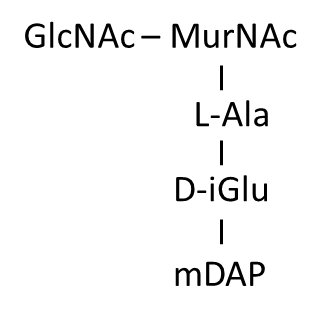

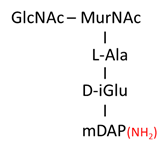

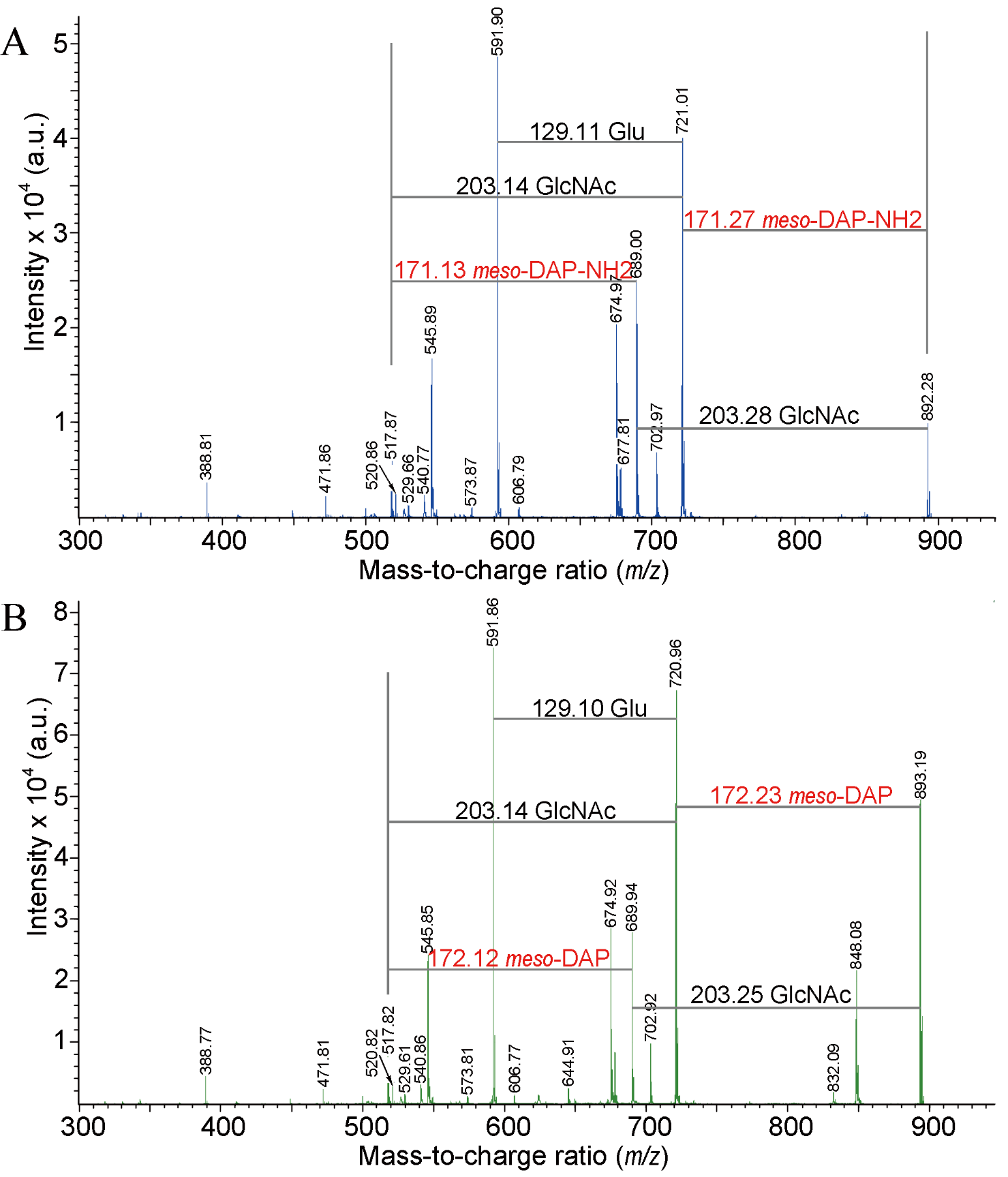
Supplementary Figures

Figure S1. The disaccharide tripeptides contained in (A) peak b of *WT/pIMK2* (Fig. 6) and (B) peak 1 of *asnB/pIMK2* (Fig. 6) were analyzed with MS-MS analysis. Fragmentation of the parental ion at m/z 892.21 for the upper panel and 893.19 for the lower panel. The inferred ions obtained by fragmentation of peptidic or glycosidic bonds and their corresponding m/z value are indicated.
